# Promoting Psychological Well-Being Through an Evidence-Based Mindfulness Training Program

**DOI:** 10.3389/fnhum.2019.00237

**Published:** 2019-07-10

**Authors:** Yi-Yuan Tang, Rongxiang Tang, James J. Gross

**Affiliations:** ^1^Department of Psychological Sciences, Texas Tech University, Lubbock, TX, United States; ^2^Department of Psychological and Brain Sciences, Washington University in St. Louis, St. Louis, MO, United States; ^3^Department of Psychology, Stanford University, Stanford, CA, United States

**Keywords:** emotions, psychological well-being, IBMT, mindfulness, bodifulness, quality of life, anterior cingulate cortex, striatum

## Abstract

Psychological well-being is a core feature of mental health, and may be defined as including hedonic (enjoyment, pleasure) and eudaimonic (meaning, fulfillment) happiness, as well as resilience (coping, emotion regulation, healthy problem solving). To promote psychological well-being, it is helpful to understand the underlying mechanisms associated with this construct and then develop targeted and effective training programs. In this perspective article, we discuss key components and potential brain-body mechanisms related to psychological well-being and propose mindfulness training as a promising way to improve it. Based on a series of randomized controlled trial (RCT) studies of one form of mindfulness training in adolescents and adults, the integrative body-mind training (IBMT), we use IBMT as an exemplar to provide research evidence of the positive effects of mindfulness training on psychological well-being. We focus on one of the mechanisms by which IBMT enhances psychological well-being—the interaction between mind (mindfulness) and body (bodifulness)—which involves both the central nervous system (CNS) and the autonomic nervous system (ANS). We also highlight the role of brain self-control networks, including the anterior cingulate cortex/prefrontal cortex (ACC/PFC), in improving psychological well-being. We suggest that mindfulness training may be a promising program that promotes the synergistic engagement of mind and body to achieve the goals of enhancing psychological well-being.

## Psychological Well-Being and Health

The importance of mental health has been increasingly emphasized in recent decades as public awareness and understanding grow. Mental health is now understood to involve both the absence of mental illness and the presence of psychological well-being. Psychological well-being is a complex construct that concerns optimal psychological functioning and experience. It may be defined as including hedonic (enjoyment, pleasure) and eudaimonic (meaning, fulfilment) happiness as well as resilience [coping, emotion regulation, healthy problem solving; Gross and Munoz, 1995; Ryff, 1995; Ryan and Deci, 2001; Community Translational Science Team (CTST), 2016; NIH Report, 2018]. Elements of psychological well-being include a sense of balance in emotion, thoughts, social relationships, and pursuits [Brown and Ryan, 2003; Community Translational Science Team (CTST), 2016; Feller et al., 2018; NIH Report, 2018], which necessitates active engagement of self-control processes such as emotion regulation. Emotion regulation is defined as the processes by which we influence which emotions we have, when we have them, and how we experience and express them (Gross, 1998).

Accumulating evidence has supported a causal relationship between greater psychological well-being and better overall health and improved disease-specific outcomes (Ong, 2010; Diener and Chan, 2011; DeSteno et al., 2013; Kok et al., 2013; Cohen et al., 2016). For example, changing states of well-being by increasing positive emotion and decreasing negative emotion result in salutary physiological/biological changes (e.g., inflammation, immune functioning), and contributes to diverse positive health outcomes (e.g., cardiovascular health; Kiecolt-Glaser et al., 2002; Howell et al., 2007; Diener and Chan, 2011; Feller et al., 2018).

## Mindfulness and Psychological Well-Being

Several systematic reviews and meta-analyses have suggested that psychological well-being can be enhanced by interventions such as mindfulness training (Brown and Ryan, 2003; Hutcherson et al., 2008; Weinstein et al., 2009; Hofmann et al., 2011; Goyal et al., 2014; Kong et al., 2016; Garland et al., 2017; McConville et al., 2017; Feller et al., 2018). One particular focus has been Integrative body-mind training (IBMT), which shares key components with other forms of mindfulness training, such as a systematic training of attention and self-control with an attitude of acceptance and openness to present experiences (Tang et al., 2007, 2015; Tang, 2017).

In a series of randomized controlled trials (RCTs), IBMT has shown multiple positive effects on psychological well-being and health, including increased self-control and positive emotions, and decreased negative emotions and stress hormones (Tang et al., 2007, 2015; Ding et al., 2014). In one RCT, young adults were assigned randomly to an IBMT or a relaxation training (RT) group for five sessions of brief training (20 min per session). Compared to those in RT, IBMT participants showed greater improvement (from baseline to post-training) of performance in executive control (an index of self-control). IBMT participants also had lower levels of negative affect and higher levels of positive affect (Tang et al., 2007; Ding et al., 2014). In addition, IBMT participants also showed decreased stress hormone cortisol and increased immune reactivity (secretory Immunoglobulin A; Tang et al., 2007). Longer training (e.g., 20 sessions) in IBMT reduced basal stress level of cortisol and increased basal immune function, suggesting better health outcomes (Tang, 2017). To test the generalizability of these IBMT findings, we used the same RCT design in older adults and adolescents and detected similar effects on psychological well-being and health (Tang, 2009, 2017; Tang et al., 2014). In particular, following long-term IBMT practice, older adults showed significantly higher ratings in overall score of quality of life, including physical, psychological, independence and social relationships using WHOQOL-100 (Tang, 2017). Taken together, findings suggest IBMT has positive effects on psychological well-being and health.

Despite these promising findings (Chambers et al., 2009; Hölzel et al., 2011b), neurobiological studies of mindfulness training directly relating behavioral changes to brain functional activity changes remain sparse (Tang et al., 2015; Fox et al., 2016). With regard to psychological well-being, functional neuroimaging studies have yet to demonstrate a straightforward relationship between brain and behavioral improvement following mindfulness training. Nonetheless, there has been some indirect evidence showing that mindfulness capacity, a trait that is often increased after mindfulness training, can modulate neural responses to emotion-related stimuli and influence affective processing (Frewen et al., 2010; Brown et al., 2013). While correlating behavioral outcomes and alterations in brain activity and functional connectivity is somewhat challenging, given the modest sample size of typical neuroimaging studies of mindfulness training, some encouraging findings have illustrated that brain structural changes are related to improved behavioral outcomes. For instance, our RCT study on IBMT has demonstrated that improvements in white matter connectivity in anterior cingulate cortex (ACC) and PCC are correlated with enhanced positive emotion, suggesting a putative neural mechanism that underlies improvement in psychological well-being (Tang et al., 2012). Likewise, one study showed that compared to waitlist control, 2 months of mindfulness-based stress reduction induced gray matter reduction in the amygdala, which was correlated with decreased stress, suggesting an improvement in psychological well-being (Hölzel et al., 2011a; Davidson and McEwen, 2012). Together, these findings provide some evidence that mindfulness training may enhance psychological well-being through influencing brain structural plasticity. However, future investigation should focus on establishing a direct relationship between brain functional changes and behavioral improvement following mindfulness training in RCT design with a large sample size.

## How Integrative Body-Mind Training (IBMT) Works

To design effective interventions for promoting psychological well-being, we need to understand how mindfulness training such as IBMT works. Our previous work has shown increases in functional and structural plasticity of self-control networks—ACC/prefrontal cortex (PFC) and striatum support IBMT effects (Tang et al., 2009, 2010, 2012, 2015; Tang, 2017). For instance, in an RCT study with 40 college students randomly assigned to either an IBMT or RT group (20/group), the IBMT group (not RT group) had a significant cerebral blood flow increase in the (ACC; BA25, BA32), adjacent medial PFC (mPFC; BA10) and insula after a five-session training. The group × session interaction was significant for BA25 and BA10, respectively (Tang, 2017). Another RCT (IBMT vs. RT) with a large sample size also showed the stronger subgenual and adjacent ventral ACC activity and striatum activity following a five-session training in IBMT (Tang et al., 2009). These findings suggest that brief IBMT changes brain activity and functional connectivity in the ACC (Tang, 2017).

Does longer IBMT practice induce brain structural plasticity? To test this hypothesis, we randomly assigned 45 college students into IBMT or RT groups (23:22) and delivered a 20-session training within a 4-week period (30 min per session, ~10 h in total). Using diffusion tensor imaging (DTI), we found significant increases in fractional anisotropy (an index of the white matter integrity and efficiency) in the ventral and dorsal corona radiata, an important white matter tract connecting the ACC to other structures (Tang et al., 2010). These changes were found in a band of white matter tracts connecting the ACC to striatal and cortical areas (Tang et al., 2010, 2012, 2015). To examine the time-course of white matter plasticity following IBMT, in another RCT, IBMT or RT groups received 10–20 sessions of training within 2–4 weeks. After 2-weeks of IBMT, the structural changes were mainly in axial diffusivity (an index of axonal density), while after 4-weeks both axial diffusivity and radial diffusivity (related to myelination) were improved (Tang et al., 2012; Tang, 2017). These findings indicated that IBMT induced functional and structural plasticity of self-control networks and fit well with meta-analyses of mindfulness effects on functional and structural changes (Hölzel et al., 2011b; Tang et al., 2015).

## The Role of Body and Mind

In IBMT practice, cooperation between body and mind is emphasized in facilitating and achieving a meditation state. We thus hypothesized that one key mechanism of IBMT involves the interaction of central nervous system (CNS, brain) and autonomic nervous system (ANS, body; Tang et al., 2007, 2009, 2015). To test the hypothesis that body (physiology) and mind (brain) interaction and balance are crucial to the observed effects of IBMT, in one RCT, one group of college students was randomly assigned to experimental (IBMT) and control (RT) conditions, and received brain imaging (cerebral blood flow) and physiological measures, whereas another group of college students was randomized into IBMT and RT conditions, but underwent EEG with physiological measures. To monitor ANS activity at rest before, during, and after five 30-min sessions of IBMT or RT, in both conditions, the physiological measures included respiratory rate and amplitude, heart rate, and skin conductance response (SCR; Tang et al., 2009).

During and after the 5-session training, the IBMT group showed lower chest respiratory rate, heart rate, and SCR, but greater belly respiratory amplitude than the RT control (Tang et al., 2009), suggesting greater parasympathetic regulation of ANS. High-frequency heart rate variability (HF-HRV) is related to parasympathetic activity of the ANS and ventral ACC activation often correlates with HF-HRV, suggesting ACC regulation of parasympathetic autonomic activity (Kubota et al., 2001; Matthews et al., 2004). Compared to the same amount of RT, we also detected increased HF-HRV and frontal midline ACC theta power, suggesting greater involvement of the ANS (especially parasympathetic activity) during and after IBMT. Brain imaging showed stronger subgenual and vACC activity following IBMT and frontal midline ACC theta also correlated with HF-HRV, suggesting control by the ACC over parasympathetic activity. These findings indicate that after brief training, the IBMT shows improved ANS regulation through the self-control system such as ACC compared to the RT. This enhancement probably reflects training in the coordination and balance of body and mind by IBMT.

**Figure 1:**
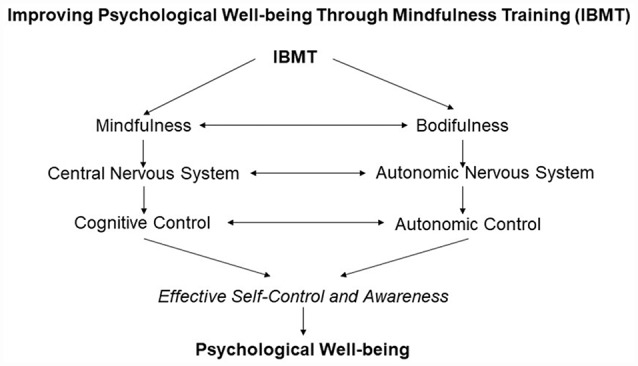
Integrative body-mind training (IBMT) and psychological well-being.

As shown in Figure 1, IBMT improves psychological well-being through mindfulness and bodifulness that mainly strengthen self-control ability and related CNS (i.e., ACC/mPFC and striatum) and ANS systems (i.e., parasympathetic activity; Tang et al., 2009, 2010, 2012, 2015; Kong et al., 2015). In the mindfulness field, mind or thought control is often emphasized, but the role of the body is often ignored (Kerr et al., 2013; Tang, 2017). Bodifulness refers to the gentle adjustment and exercise of body posture with a full awareness, in order to achieve a presence, balance, and integration in our bodies. For instance, bodifulness mainly involves implicit processes such as visceral or interoceptive awareness regulated by ANS. Autonomic control requires less effort and is mainly supported by the ACC/mPFC and striatum (Critchley et al., 2003; Naccache et al., 2005; Jones et al., 2015; Tang et al., 2015; Tang, 2017).

In IBMT practice, mind–body coordination and interaction are emphasized and thus significantly facilitate the training outcomes (Tang et al., 2007, 2015; Tang, 2009, 2017). Full awareness and presence of the body (bodifulness) could facilitate the mindfulness process, consistent with the literature that body posture and state affect mental processes such as emotional processing, the retrieval of autobiographical memories, and cortisol concentrations (Hennig et al., 2000; Dijkstra et al., 2007; Niedenthal, 2007; Huang et al., 2011). In early stage, mindfulness requires conscious cognitive control with effort and is supported by the dorsal lateral PFC and parietal cortex but over time, it may well involve less effort when the practice becomes more skillful, which is supported by the ACC and striatum (Tang, 2017). Cognitive control (termed as doing state) and autonomic control (termed as being state) are both key components of self-control supported by the practice and interaction of mindfulness and bodifulness, which may drive behavior and habit formation effectively (Tang et al., 2015; Tang, 2017). Taken together, if interventions such as IBMT target increases in psychological well-being through the engagement of both body and mind, effectiveness may be enhanced.

## Conclusions

A growing literature supports the idea that there is an important relationship between psychological well-being and mental and physical health in both adolescents and adults (Ong et al., 2011; DeSteno et al., 2013). IBMT has been shown to improve psychological well-being, and appears to do so *via* changes in self-control that are reflected in changes in both the central (brain/mind) and the autonomic (body/physiology) nervous systems. In particular, IBMT changes the state of body and mind to lead to positive outcomes in emotion, cognition, and behavior. IBMT is a paradigmatic case of how it is possible to use an evidence-based intervention that targets brain, physiology, and behavior to achieve the goals of psychological well-being and human flourishing.

In this perspective piece, we have focused on the self-control networks supporting psychological well-being following IBMT, but it should be noted that the reward system supporting motivation and positive emotion also closely links to psychological well-being (Tang et al., 2009, 2015; Tang, 2017). Future work should explore the interaction between self-control and reward systems that improve and optimize psychological well-being and mental and physical health. Moreover, different interventions may target different brain networks, and it’s important to understand how different types of training programs differentially impact different brain systems and how different people might preferentially benefit from one type of intervention vs. another (Tang et al., 2015, 2016).

## Data Availability

No datasets were generated or analyzed for this study.

## Author Contributions

Y-YT, RT and JG contributed to manuscript writing.

## Conflict of Interest Statement

The authors declare that the research was conducted in the absence of any commercial or financial relationships that could be construed as a potential conflict of interest.
